# PKA-driven *SPP1* activation as a novel mechanism connecting the bone microenvironment to prostate cancer progression

**DOI:** 10.1038/s41388-025-03511-z

**Published:** 2025-08-02

**Authors:** Pablo Sanchis, Agustina Sabater, Julia Lechuga, Jimena Rada, Rocio Seniuk, Gaston Pascual, Mora Gatti, Juan Bizzotto, Peter D. A. Shepherd, Jun Yang, Javier Cotignola, Elba Vazquez, Joaquin Mateo, Pia Valacco, Estefania Labanca, Christopher Logothetis, Geraldine Gueron, Nicolas Anselmino

**Affiliations:** 1 Department of Genitourinary Medical Oncology and The David H. Koch Center for Applied Research of Genitourinary Cancers, The University of Texas MD Anderson Cancer Center, Houston, TX USA; 2 Laboratorio de Inflamación y Cáncer, Departamento de Química Biológica, Facultad de Ciencias Exactas y Naturales, Universidad de Buenos Aires, Buenos Aires, Argentina; 3 CONICET-Universidad de Buenos Aires, Instituto de Química Biológica de la Facultad de Ciencias Exactas y Naturales (IQUIBICEN), Buenos Aires, Argentina; 4 Universidad Argentina de la Empresa (UADE), Instituto de Tecnología (INTEC), Buenos Aires, Argentina; 5 Prostate Cancer Research Group, Vall d’Hebron Institute of Oncology (VHIO), Vall d’Hebron University Hospital, Barcelona, Spain; 6 Department of Medical Oncology, Vall d’Hebron University Hospital and Vall d’Hebron Institute of Oncology (VHIO), Barcelona, Spain

**Keywords:** Bone metastases, Cancer microenvironment

## Abstract

Prostate cancer (PCa) bone metastasis (BM) poses a significant clinical challenge due to the heterogeneity of treatment responses and patient outcomes. In this study, we examined the role of Protein Kinase A (PKA) signaling in modulating the expression of osteopontin (*SPP1*/OPN), a protein associated with poor prognosis, within a subset of PCa BM patients. By integrating multi-omics results we identified a novel mechanism in which bone-derived type-I collagen (Col1a1) and fibronectin (Fn1) stimulate *SPP1* expression in PCa cells through the activation of PKA signaling. This bone-induced regulation of *SPP1* was confirmed both in vitro, using PCa-bone co-culture systems (PC3 or C42B/MC3T3 cell lines), and in vivo, using cell lines’ engraftments and patient-derived xenografts (PDX) grown intrafemorally. Importantly, clinical data from longitudinal patient samples revealed that treatment with enzalutamide, an androgen receptor (AR) inhibitor, led to an increase in PKA signaling and corresponding *SPP1* expression in a subpopulation of patients, highlighting the relevance of the PKA/*SPP1* axis in disease progression under AR-targeted therapies. Overall, we underscored the critical role of the bone microenvironment in influencing PCa progression, pointing out to *SPP1*/OPN as a biomarker for identifying tumors with active PKA signaling, which could serve to manage resistance to AR-directed treatments.

## Introduction

Prostate cancer (PCa) is one of the most prevalent malignancies in men, and its metastatic progression poses significant challenges for clinical management [1, 2], with bone as the main metastatic site. The molecular underpinnings of PCa, particularly in the context of bone metastasis (BM), remain a critical area of investigation.

PCa progression is dominated by constant tumor adaptation [3] The acquisition of resistance to androgen deprivation therapy coincides with PCa BM in most cases [3], indicating the presence of bone microenvironment (BME)/epithelial interactions that drive organ-specific progression [4–6].

*SPP1*/OPN (secreted phosphoprotein 1/osteopontin), a protein initially characterized for its role in bone physiology, has been tightly associated with PCa and breast cancer (BCa) pathogenesis [7], and has been implicated in other tumors types. During bone resorption, OPN facilitates osteoclast attachment to the matrix and plays a role in osteogenesis and crystal size regulation during bone mineralization [8]. OPN’s interaction with αvβ3 integrin on osteoclasts reduces cytosolic calcium, inducing podosome formation and bone resorption, while promoting RANKL expression and osteoclast migration [9]. In tumoral cells, OPN binding to αvβ3 and CD44 enhances adhesion, migration, and invasion in bone metastases, activating signaling pathways like PKCα/c-Src/IKK/NF-κB, leading to COX-2 expression, PGE2 production, MMP-2 activation, and angiogenesis [7]. In the context of bone metastasis, *SPP1*/OPN is also involved in osteoclast adhesion and bone resorption, raising the possibility that tumor-derived OPN may contribute to metastatic colonization and survival in the bone niche [7]. Clinically, elevated OPN levels correlates with tumor stage and poor survival in BCa BM [7]. High plasma OPN has also been proposed as a biomarker of metastatic castration-resistant PCa (CRPC) [10]. Indeed, a systematic review and meta-analysis indicates that OPN levels positively correlates with PCa Gleason grade, stage, and metastasis, whereas inversely correlates with overall and relapse free survival [11]. However, the regulatory mechanisms underlying *SPP1*/OPN increased expression remain unknown for PCa and its induction seems to occur in only a fraction of bone-metastatic PCa [12], suggesting a particular molecular PCa subgroup. Given the heterogeneity of PCa, it is crucial to identify the molecular hubs governing *SPP1*/OPN expression in this subpopulation to improve disease management.

Based on the heterogeneity of bone-metastatic PCa we hypothesize that bone-derived cues activate protein kinase A (PKA) signaling in PCa cells in a subpopulation of patients, thereby driving *SPP1*/OPN expression. By integrating patient’ datasets with in vitro and in vivo experimental models we identified a novel, clinically relevant molecular axis, characterized by bone derived collagen and fibronectin that stimulate *SPP1*/OPN expression via PKA activation, which may promote disease progression and resistance to androgen receptor (AR) pathway-targeted therapies. Our findings suggest that *SPP1*/OPN holds potential as a biomarker for PCa progression to bone through PKA activation.

## Materials/subjects and methods

### Cell lines and co-culture system

PCa cells were chosen based on their AR status and sensitivity to AR-targeted therapies. Human PC3 and C42B (AR− and AR+ metastatic PCa cell lines, respectively); and murine MC3T3 (pre-osteoblastic cell line) were purchased from the American Type Culture Collection and bi-weekly tested for *Mycoplasma* using Mycolor One-Step Mycoplasma Detector (Vazyme, China). PCa cells were co-cultured with MC3T3 cells as previously described [13, 14] using an in vitro bio-compartment culture system as it mimics paracrine interactions between tumor cells and bone progenitors. PCa cells were seeded at a density of 100,000 cells/insert in 6-well plate cell-culture inserts (0.4 mm pore; Falcon/Becton Dickinson Labware, Franklin Lakes, USA). MC3T3 cells were seeded in 6-well culture plates at a density of 200,000 cells/well. After 24 h, inserts containing PCa cells were placed into tissue-culture plates containing MC3T3 cells. The two different cell lines shared the culture medium but were not in physical contact. Co-culturing of PCa cells with MC3T3 was performed with α-MEM, supplemented with 2% FBS for 24 h and cells were harvested separately. As control, each cell line was grown alone. Conditioned media (CM) were collected and stored at −80 °C. At least 3 experimental replicates were performed for each experimental approach using the co-culture system.

### RNA sequencing and differential gene expression analysis in vitro

RNA was extracted and sequenced from MC3T3 cells cultured alone or co-cultured with PC3 cells as previously published [14]. After mapping RNA-seq reads to the mouse GRCm38 reference genome using HISAT2 [15], differential expression analysis was performed using the GFOLD algorithm (c = 0.01) [16] for MC3T3 cells co-cultured with PC3 cells compared with MC3T3 alone. RNA pooled from 5 independent experiments/conditions was used for the RNA-seq.

### RT-qPCR

Complementary DNAs were synthesized and used for real-time PCR as previously described [14]. *PPIA* was used as the internal reference gene. Supplementary Fig. 1 shows *PPIA* variability among different conditions evaluated. The obtained data were analyzed using the method of 2^−ΔΔCT^ [17]. Primer sequences: *PPIA_*forward: 5’-GGTATAAAAGGGGCGGGAGG-3’, *PPIA_*reverse: 5’-CTGCAAACAGCTCAAAGGAGAC-3’, *SPP1_*forward: 5’-AGCCTTCTCAGCCAAACGC-3’, *SPP1_*reverse: 5’-TGGAAGGGTCTGCTTTTCCTC-3’, *PRKACB*_forward: 5’-CCAGGTCACAGACTTTGGGT-3’, *PRKACB*_reverse: 5’-GCACTCCTAATGCCCACCAA-3’, *PRKACA*_forward: 5’- AGTACCTGGCCCCTGAGATTA-3’, *PRKACA*_reverse: 5’- AAGTGGGAAGGGAAGCGCA-3’, *KLK3*_forward: 5’- TGAACCAGAGGAGTTCTTGAC, *KLK3*_reverse: 5’-CCCAGAATCACCCGAGCAG-3’.

### Western blotting

Immunoblot was carried out as previously described [18] using the following antibodies: anti-OPN (cat.#22952-1-AP; Proteintech; Rosemont, USA; 1:2000), anti–α-tubulin (cat.#3873; Cell Signaling; Danvers, USA; 1:2000), anti-AR (cat. #BSB6074, Bio SB, Santa Barbara, USA; 1:1000), and anti-mouse and anti-rabbit antibodies (cat.#7076S and cat.#7074S, respectively; Cell Signaling, Danvers, USA; 1:5000).

### Secretome analysis

Proteomics analysis by Liquid Chromatography-Electrospray Ionization-Tandem Mass Spectrometry (LC ESI-MS/MS) was previously performed [14] using the CM obtained from the co-culture experiments as previously published (Supplementary Methods) [18]. The MS data were analyzed using Proteome Discoverer software (version 2.1.1.21; Thermo), employing the Sequest search engine [19], against *Homo sapiens* and *Mus musculus* protein sequences from the Uniprot database. Search parameters included trypsin digestion with 1 miscleavage, fixed carbamidomethylation of cysteines and oxidation of methionines (variable) as post-translational modifications. The search allowed a parent ion tolerance of 10 ppm and a fragment mass tolerance of 0.05 Da and. Peptides were identified with a false discovery rate of less than 1%, calculated using a concatenated decoy database. OPN–secretome interactions were evaluated using STRING [20] (accessed May 2023).

### Adhesion assay

Cell adhesion assays were performed over a matrix of inorganic calcium crystals mimicking bone (Corning #3989, USA). Wells were coated with CM of PC3 cells, PC3/MC3T3 co-culture, and MC3T3 cells. For this, 200 µl of CM were added to each corresponding well and incubated for 1 h. The CM was removed and 50 000 PC3 cells were seeded with 200 µl of the respective CM or fresh culture medium. After 1 h, the medium was removed and two washes with PBS were performed to remove non-adherent cells. Adhered cells were fixed with 10% methanol/10% acetic acid/water for 20 min at room temperature and stained with 0.5% crystal violet for 10 min. Crystal violet was extracted with 100 μl of 10% acetic acid for 10 min, and absorbance was quantified (600 nm).

### PKA modulation

PKA activity was inhibited using H89 (B1425, Sigma-Aldrich, St. Louis, USA), 10 µM for 3 h, as previously published [14]. To induce PKA activity, we used forskolin (FK; Cat. AAJ63292MA, Fisher Scientific, Hampton, USA) 1 µM for 30 min and 24 h.

### Transfection and dihydrotestosterone treatment

80,000 PC3 cells were plated in 6-multiwell plates for 24 h and transfection with expression vectors pcDNA3 (Invitrogen, Waltham, USA), pcDNA3-AR [21] and the reporter vector PSA-Luc [22] was done at the following day using Lipofectamine 3000 (Invitrogen, Waltham, USA) in charcoaled FBS using 2.5 µg of plasmid. After 24 h, PC3 cells were treated with FK 1 µM and dihydrotestosterone (DHT) 10 nM for 24 h.

### Immunofluorescence

PKA activity was measured by immunofluorescence using an antibody that recognizes phosphorylated PKA (p-PKA) substrates and confocal microscopy as published elsewhere [18]. We used anti-phospho-PKA substrate antibody (cat.#9624; Cell Signaling; Danvers, USA; 1:200) and fluorescent secondary antibody Alexa Fluor 647 anti-rabbit (Invitrogen, Waltham, USA; 1:3000). Cells were counterstained with DAPI and imaged by confocal microscopy (Olympus Fluo View FV 1000 microscope, Olympus 60×/1.20 NA UPLAN APO) or epifluorescence microscope.

### Cell viability

C42B cells (3000 cells/well; 96-multiwell plate) were treated with FK 1 µM for 24 h or 30 min, prior to enzalutamide (Sellekhem, Houston, USA) 30 or 50 µM exposure for 96 h. DMSO was used as vehicle. Cell viability assay was performed using the CellTiter-Glo kit (Promega, Madison, USA).

### Ethics approval

All practices involving animals were approved by the Institutional Animal Care and Use Committee of The University of Texas MD Anderson Cancer Center (MDACC), under the regulation of the Animal Welfare Committee and conform to the NIH Policy on Human Care and Use of Laboratory Animals (protocol: #00001091-RN03). The number of animals used was estimated based on previous results [23, 24].

### MDA PCa 118b and MDA PCa 183-A patient-derived xenografts

Intrafemoral (*i.f*.) patient-derived xenografts (PDXs) MDA PCa 118b and MDA PCa 183-A were previously developed and processed from the respective bone marrow aspirates of men with metastatic adenocarcinoma [23, 25, 26]. These models were chosen as they originate from bone metastases and reflect distinct AR signaling contexts—enabling exploration of castration resistance and bone colonization [26, 27]. Cells (1 × 10^6^) derived from MDA PCa 183 and 118b PDX were injected *i.f*. into the distal end of right femurs of 6-to 8-wk-old male CB17 *SCID* mice. Left legs were sham-injected, non-tumor-bearing controls [14, 26]. After harvesting, bones were flash frozen. Each PDX was grown *i.f*. in 5 mice.

### RNA-seq of intrafemoral tumors

RNA was extracted from fresh frozen tissues at the Biospecimen Extraction Facility (MDACC) using the QIAGEN RNeasy Kit (Hilden, Germany). Stranded mRNA libraries were prepared using the KAPA Stranded mRNA-seq Kit. Briefly, 250 ng of total RNA was captured using magnetic Oligo-dT beads and fragmented using heat and magnesium. First-strand synthesis was performed using random priming followed by second-strand synthesis with the incorporation of dUTP into the second strand. The ends of the resulting double-stranded cDNA fragments were repaired, 5’-phosphorylated, 3’-A tailed, and Illumina-specific indexed adapters were ligated. The products were purified and enriched for full-length library with nine cycles of PCR. The strand marked with dUTP was not amplified, resulting in a strand-specific library. The libraries were quantified using the Qubit dsDNA HS Assay Kit (Thermo Fisher Scientific, Waltham, USA) and assessed for size distribution using the 4200 TapeStation High Sensitivity D1000 ScreenTape (Agilent Technologies). Libraries were then multiplexed, 48 libraries per pool. The library pool was quantified and sequenced at the Advanced Technology Genomics Core (MDACC), in one lane of the NovaSeq6000 S2-Xp flow cell using the 100-nt paired-end format. Sequencing data were processed using an established in-house bioinformatics pipeline at the MDACC Department of Genomic Medicine. Reads were aligned to the human (hg19) and mouse (mm10) reference genome using STAR [28]. HTSeq [29] was used for mapping gene count quantification. We employed species-specific sequencing alignment pipelines to distinguish between human (tumor-derived) and mouse (stroma- or bone-derived) transcripts. Fragments Per Kilobase of transcript per Million mapped reads (FPKM) method was used to normalize human or murine transcripts separately.

### PC3 subcutaneous and intrafemoral xenografts

PC3 cells were injected either subcutaneously (*s.c*.) or *i.f*. (5 × 10^4^ cells) as described above. Tumor growth was monitored weekly by caliper or x-ray/µCT, respectively. PC3 tumors and tumor-bearing bones were harvested 1 month after implantation. After harvesting, femurs were fixed in formalin, decalcified (*i.f*.) and embedded in paraffin for histological analysis.

### Castration experiments

MDA PCa 183 PDX or C42B-luc cells were implanted *i.f*. into the distal end of right femurs of 6- to 8-wk-old male CB17 *SCID* mice (1 × 10^6^ cells and 5 × 10^4^, respectively), and monitored by x-ray/µCT and in vivo imaging system (IVIS), respectively. After a month, mice were randomized into sham-castrated (control) or surgically castrated groups. Tumor-bearing bones were harvested 7 weeks (MDA PCa 183) or 4 weeks (C42B) after castration, fixed in formalin for 48 h, decalcified in EDTA pH 7.4 for 5 days, and embedded in paraffin for further histological analysis.

### Immunohistochemistry

Blinded immunohistochemistry (IHC) analyses of OPN and p-PKA substrate expression in *s.c*. tumors, tumor-bearing bones castrated or sham-castrated were performed. Sections were stained with anti-OPN antibody (cat.#83341 1:500; Proteintech; Rosemont, USA), and anti-p-PKA substrate antibody (cat.#9624; Cell Signaling; Danvers, USA; 1:200) described elsewhere [30].

### Datasets

The GSE74685 [31], SU2C-PCF [32], GSE32269 [33], and Westbrook et al. [34] datasets were selected because they offer transcriptomic data from different PCa progression sites, including bone metastasis. GSE74685 (171 samples from primary [*n* = 14] or metastatic PCa tumors) [31]; SU2C-PCF dataset (444 metastatic samples from CRPC tumors) [32]; Westbrook et al. (paired biopsies from 21 men with metastatic CRPC who had a tissue biopsy performed pre and post [at the time of progression] treatment with enzalutamide) [34]; GSE32269 (22 primary PCa and 29 BM) [33]. For those analyses where we divided the cohort into two groups based on *SPP1* mRNA expression, the mean of expression was used as the cutoff value.

### Ingenuity pathway analysis

Ingenuity Pathway Analysis [35] (IPA, QIAGEN Inc., Germantown, USA) was used to study upstream regulators. This software utilizes a comprehensive knowledge base of curated biological interactions and functional annotations. It enables the identification of key regulators, pathways, and biological processes relevant to the experimental data. By mapping the data into known biological networks, IPA provides insights into the potential upstream regulators and their impact on the observed molecular changes. This approach facilitates a deeper understanding of the underlying biological mechanisms.

### Statistical analysis

Wilcoxon, Kruskal–Wallis, t test, or ANOVA tests were used to assess *SPP1* expression across tissue samples/conditions and plotted in GraphPad Prism software (La Jolla, USA). The specific statistical analysis performed for each analysis is described in the respective legend to figure. At least three independent experiments were performed for each in vitro approach, and two to three technical replicates per independent experiment. Differential gene expression in the GSE74685 was performed using Limma package [36] in R and Benjamini-Hochberg correction was used to calculate the False Discovery Rate (“adjusted *P* value”). Gene Ontology (GO) classification was performed using the clusterprofiler [37] and enrichplot [38] packages in R. Statistical significance was set at *P* < 0.05.

Additional information about the methodology can be found in the Supplementary Methods section.

## Results

### The BME drives *SPP1* transcriptional activation

**Fig. 1:**
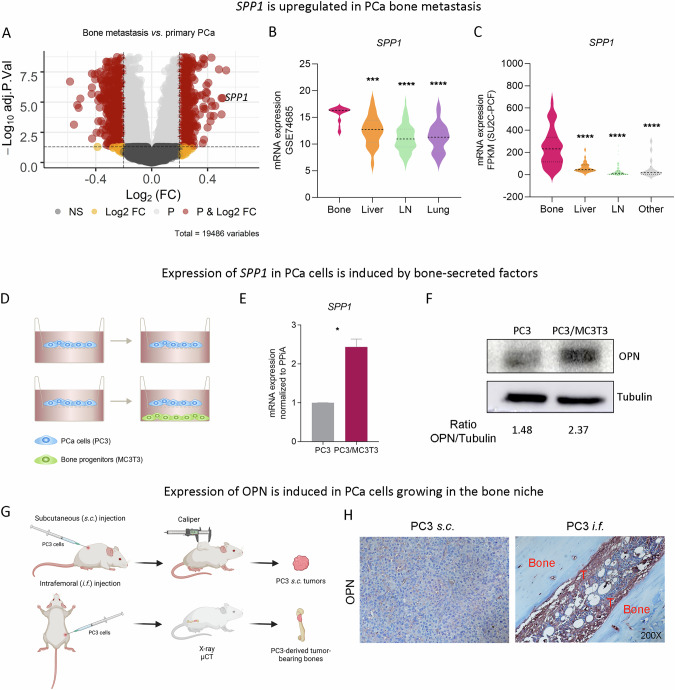
*SPP1* is induced in PCa bone metastasis. **A** Volcano plot depicting differential gene expression analysis of the GSE74685 dataset comparing bone metastases (*n* = 20) *vs*. primary PCa (*n* = 14) samples. Significant differential expression (*P* < 0.05, |Log_2_ fold change|>0.2) is represented for each comparison as cherry dots. Limma package in R was used to calculate statistical significance with a Benjamini–Hochberg correction (adjusted *P* value). NS not significant, Log_2_FC Log_2_ fold change. Dark gray dots: |Log_2_ fold change| < 0.2 and *P* > 0.05; yellow dots: |Log_2_ fold change| > 0.2 and *P* > 0.05; light gray dots: |Log_2_ fold change| < 0.2 and *P* < 0.05; cherry dots: |Log_2_ fold change| > 0.2 and *P* < 0.05. **B**, **C** Violin plots showing gene expression levels of *SPP1* in PCa bone metastasis and different metastatic sites from the GSE74685 (bone (*n* = 20), liver (*n* = 21), lymph node (*n* = 69) and lung (*n* = 22)) and SU2C-PCF (bone (*n* = 83), liver (*n* = 26), lymph node (*n* = 79), and other metastasis (*n* = 14)) datasets, respectively. One-way ANOVA was used to assess statistical significance. **D** PC3 cells were cultured alone or co-cultured with bone progenitors (MC3T3) using an in vitro bicompartment, which allows cells to share the culture medium and signaling factors without physical contact, mimicking the interactions between tumor cells and the bone metastatic niche through soluble factors. Cells were seeded in their respective compartments, and after 24 h, the inserts containing PC3 cells were washed and placed in the co-culture plates with or without MC3T3 cells. On day 3, PC3 cells were harvested, and RNA was extracted for gene expression analysis. **E** Gene expression levels of *SPP1* by RT-qPCR in PC3 grown alone (PC3) and in PC3 co-cultured with MC3T3 (PC3/MC3T3). Values were normalized using *PPIA* as a reference gene and relativized to the control. Statistical significance was assessed via Wilcoxon test. Results are shown as the mean ± S.E.M. Statistical significance was set at *P* < 0.05. *P < 0.05. Three independent experiments were performed. **F** OPN expression levels in PC3 and PC3/MC3T3 assessed by Western blot relativized to tubulin. **G** Schematic representation of subcutaneous (*s.c*.) and intrafemoral (*i.f*.) implantation of PC3 cells in male CB17 SCID mice and samples subsequently processed for immunohistochemical analysis. **H** One representative photomicrograph image of PC3 tumors *s.c*. and *i.f*. sections immunostained with OPN (*n* = 5). Magnification 200X. T Tumor.

Bioinformatics analyses using the GSE74685 dataset [31] revealed *SPP1* among the top 3 upregulated genes in BM *vs*. primary PCa (Fig. 1A; Supplementary Table 1). Moreover, *SPP1* mRNA levels were significantly higher in BM compared with other metastatic sites (liver, lymph nodes, lung and other), in the GSE74685 (Fig. 1B) and SU2C-PCF [32] datasets (Fig. 1C), suggesting that the BME participates in the regulation of *SPP1* expression in PCa cells.

To understand how the dialog between PCa and bone cells modulates *SPP1* expression, we performed an indirect co-culture between PC3 and MC3T3 cell lines (Fig. 1D). A significant increase in *SPP1/*OPN mRNA (Fig. 1E) and protein (Fig. 1F) expression was observed in PC3 cells when co-cultured with bone progenitors, recapitulating what was observed in clinical samples. Of note, although qualitative, we also found OPN secreted in the conditioned media (CM) of the assessed conditions (Supplementary Fig. 2). Moreover, to better reflect the complexity of the bone microenvironment PC3 cells were implanted either *s.c*. or *i.f*. in 6- to 8-wk-old male CB17 *SCID* mice (Fig. 1G). Strikingly, OPN protein levels were induced in PC3 cells growing *i.f*. compared to *s.c*. (Fig. 1H). Thus, *SPP1*/OPN expression in PCa cells is induced by the bone niche and involves bone cell-secreted factors released during the PCa-bone crosstalk.

### Identification of bone-secreted factors regulating *SPP1* transcriptional activation

**Fig. 2:**
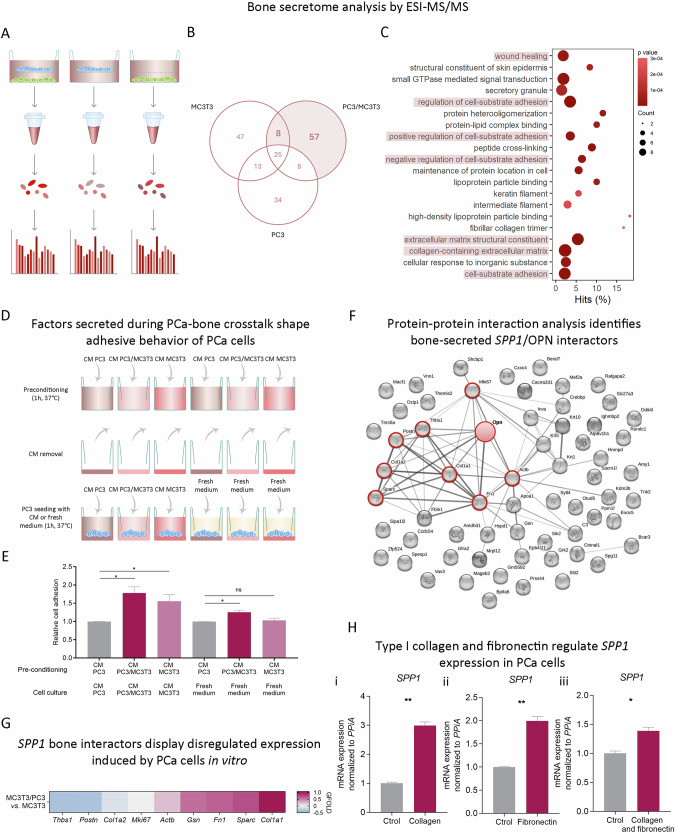
Bone-secreted factors regulate *SPP1* transcriptional activation. **A** Experimental design for secretome analysis. After co-culturing PC3 and MC3T3, the conditioned media (CM) of each condition was centrifuged and analyzed by LC ESI/MS-MS. **B** Exclusion/inclusion criteria of differential proteins represented as a Venn diagram, where the numbers correspond to the proteins detected in each CM when LC ESI/MS-MS data were contrasted with murine protein database. Proteins in the CM the co-culture also found in the CM of PC3 cells alone were excluded in the following analyses. **C** Gene Ontology categories enriched considering the 65 murine proteins identified in the PC3/MC3T3 co-culture. The intensity of the color depicts the *P* value. The size of the dots represents the number of proteins identified for each biological process. **D** Experimental design of the cell adhesion assay. Commercial wells covered with a bone-like matrix (Corning, Cat. #3988) were treated with the CM from PC3 or MC3T3 cells growing alone, or from the PC3/MC3T3 co-culture. The preconditioning media were then removed and PC3 cells were cultured in the presence of the respective CMs, or with fresh culture medium. After 1 h, cells were washed with PBS and stained with crystal violet. Crystal violet was extracted with 10% acetic acid (V/V), and quantification of absorbance at 600 nm was performed Statistical significance was assessed via Kruskal–Wallis test. **E** Bar plot representing cell adhesion levels of PC3 cells to a bone-like matrix relative to control. **F** Protein-protein association analysis between OPN and the bone-secreted factors identified by LC ESI-MS/MS using STRING. OPN is represented as a red ball. OPN interactors are represented as gray balls with cherry borders. **G** Heatmap showing differential expression (GFOLD) of genes encoding OPN-related bone-secreted proteins assessed by RNA-seq in MC3T3 cells co-cultured with PC3 cells compared to MC3T3 cells cultured alone. The color scale goes from blue (GFOLD = −0.5) to cherry (GFOLD = 1). **H** *SPP1* gene expression levels assessed by RT-qPCR in PC3 cells seeded in wells coated with type I collagen (i), fibronectin (ii) or coating mix (type I collagen and fibronectin; iii). PC3 cells seeded in untreated wells were considered controls (Ctrol). Values were normalized using *PPIA* as a reference gene and relativized to controls. Statistical significance was assessed via Wilcoxon test. Results are shown as mean ± S.E.M. Three independent experiments were performed. Statistical significance was set at *P* < 0.05. *P < 0.05, **P < 0.01.

To identify the bone-associated factors responsible for *SPP1* induction in PCa, we performed a proteomics analysis (LC ESI-MS/MS) of the CM from the co-culture experiment (Fig. 2A). We identified 65 murine proteins secreted by MC3T3 cells in the PC3/MC3T3 co-culture (Fig. 2B). Of note, by Gene Ontology we observed a significant enrichment in categories associated to cell-substrate adhesion, extracellular matrix and wound healing (Fig. 2C). Consistently, pre-conditioning a bone-mimicking matrix with PC3/MC3T3 co-culture CM significantly increased the adhesive capacity of PC3 cells compared with pre-conditioning with PC3 CM (Fig. 2D, E), further confirming the effect of the soluble factors released during the PC3/MC3T3 crosstalk in PC3 cells behavior. Interestingly, MC3T3 CM exerted this effect on PC3 adhesion only when it was used as culture media, in addition to pre-conditioning (Fig. 2D, E). These results suggest that MC3T3 secreted factors trigger PC3 cells to release molecules that enhance their adhesion to the bone matrix, consistent with tumoral *SPP1*/OPN induction.

Protein-protein association analysis between OPN and the bone secretome showed the association of OPN with Col1a1, Col1a2, Sparc, Fn1, Gsn, Actb, Postn, and Thbs1 (Fig. 2F, red filled circles). Moreover, co-culture with PCa cells modulated the expression of these OPN-associated proteins in MC3T3 cells, particularly inducing *Col1a1*, *Sparc*, *Fn1*, and *Gsn* gene expression (Fig. 2G).

Interestingly, we recently proposed a novel communication axis by which bone secreted factors like Col1a1 and Fn1 may induce metabolic rewiring in PCa cells via PKA activation [14]. Fn1 and Col1a1 are critical extracellular matrix proteins involved in bone remodeling [39] as well as key bone-released factors mediating the interaction with PCa cells [14]. Thus, we assessed the influence of these proteins on *SPP1* modulation in PCa and observed that *SPP1* levels were significantly higher in PC3 cells cultured on plates coated with type I collagen (*P* = 0.0041) (Fig. 2Hi), fibronectin (*P* = 0.0076) (Fig. 2Hii) or type I collagen and fibronectin together (*P* = 0.0331) (Fig. 2Hiii), compared with control (uncoated), evidencing the regulatory effect of Fn1 and Col1a1 on *SPP1* expression.

### Tumoral PKA activation regulates *SPP1* expression

**Fig. 3:**
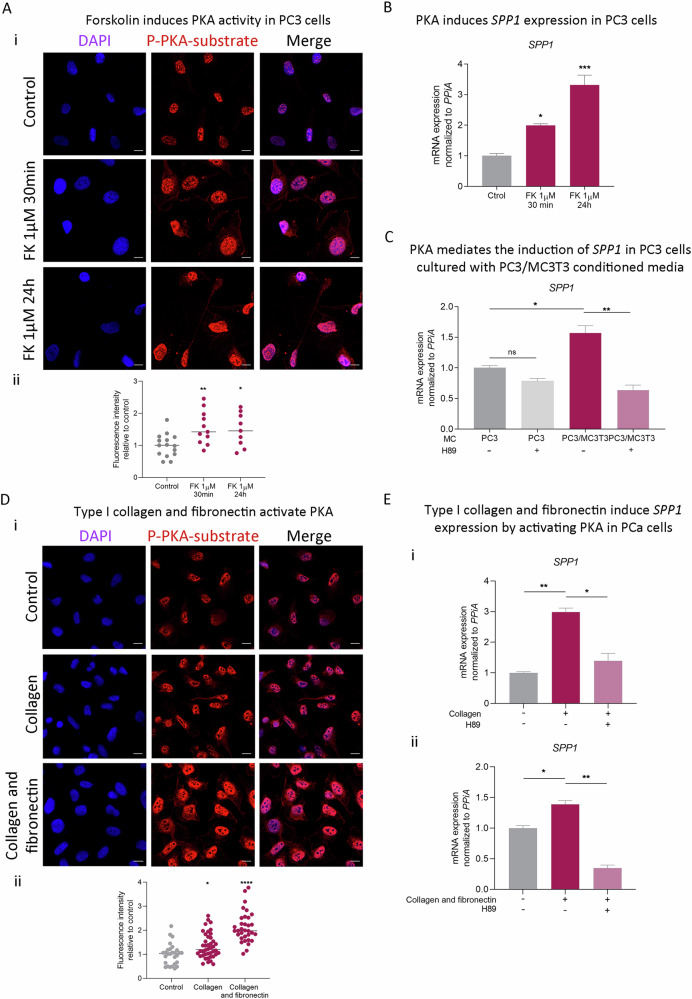
Bone-released Col1a1 and Fn1 induce *SPP1* expression through PKA activation. **A** (i) Immunofluorescence (IF) staining and confocal microscopy analysis for phosphorylated PKA (p-PKA) substrate on PC3 cells treated or not treated with forskolin (FK; 1 μM; 0.5 h or 24 h). Cell nuclei were counterstained with DAPI. (ii) Quantitative analyses of the IF. Fluorescence intensity for p-PKA substrate was determined using ImageJ. **B** *SPP1* gene expression levels assessed by RT-qPCR in PC3 cells treated or not (Ctrol) with FK 1 μM for 30 min or 24 h. Values were normalized using *PPIA* as a reference gene and relativized to controls. Results are shown as mean ± S.E.M. **C** *SPP1* gene expression levels evaluated by RT-qPCR in PC3 cells treated with the conditioned medium (CM) of PC3 grown alone or with the CM of the PC3/MC3T3 co-culture for 24 h, and with or without the addition of the PKA inhibitor, H89 (10 µM), during the last 3 h of culture. Values were normalized using *PPIA* as a reference gene and relativized to the CM of PC3. **D** (i) IF staining and confocal microscopy analysis for p-PKA substrate and (ii) fluorescence intensity quantification in PC3 cells seeded on type I collagen or type I collagen and fibronectin or control. **E** *SPP1* gene expression levels evaluated by RT-qPCR in PC3 cells cultured for 24 h in wells coated with type I collagen (i) or coating mix (ii; type I collagen and fibronectin), with or without H89 (10 µM) added during the last 3 h of culture. PC3 cells seeded in untreated wells were considered controls. Values were normalized using *PPIA* as a reference gene and relativized to controls. Kruskal–Wallis was used to evaluate statistical significance. Results are shown as mean ± S.E.M. Three independent experiments were performed. Statistical significance was set at *P* < 0.05. *P < 0.05, **P < 0.001, ***P < 0.0001.

To determine whether PKA regulates *SPP1*, we induced PKA activity using FK 1 µM (30 min or 24 h) on PC3 cells. Activity was confirmed by increased levels of phosphorylated PKA substrate (Fig. 3A). Strikingly, *SPP1* mRNA was upregulated by 2 to 3-fold (*P* < 0.05) by short or long-term FK treatment, respectively (Fig. 3B), implicating PKA in *SPP1* regulation. Consistently with the nuclear staining we observed for phosphorylated PKA substrate, we found that the transcription factor CREB, which is one of the most described PKA targets, and its associated protein, CREBP1, recognize DNA motifs within the *SPP1* promoter sequence with a similarity score higher than 0.8 (range of the similarity score: 0-1; Supplementary Fig. 3B, Supplementary Methods), highly suggestive that upon PKA activation, it phosphorylates CREB/CREBP and these in turn activate *SPP1* expression. Moreover, *SPP1* induction by the CM from the PC3/MC3T3 co-culture (Fig. 3C) was significantly (*P* < 0.01) reversed by PKA inhibition (H89 10 µM) (Fig. 3C), as well as cell adhesion to the substrate (Supplementary Fig. 4). These results confirm that bone-induced *SPP1* expression in PCa is mediated by PKA activity, and that this kinase plays a critical role in PCa cells behavior. Accordingly, the presence of collagen and fibronectin activated PKA (Fig. 3Di and ii, Supplementary Fig. 5A), while PKA inhibition reverted collagen and fibronectin–induced *SPP1* expression (Fig. 3Ei and ii, respectively, and Supplementary Fig. 5B). Of note, higher staining levels were observed in PC3 cells growing *i.f*. in mice compared with *s.c*. growth (Supplementary Fig. 5C), confirming the role of the microenvironment in activating this kinase.

These results indicate that Col1a1 and Fn1 activate PKA signaling in PCa cells, inducing *SPP1* expression. Thus, *SPP1/*OPN emerges as a potential biomarker of PKA activity.

### Correlation between PKA activity and *SPP1* expression in clinical samples

**Fig. 4:**
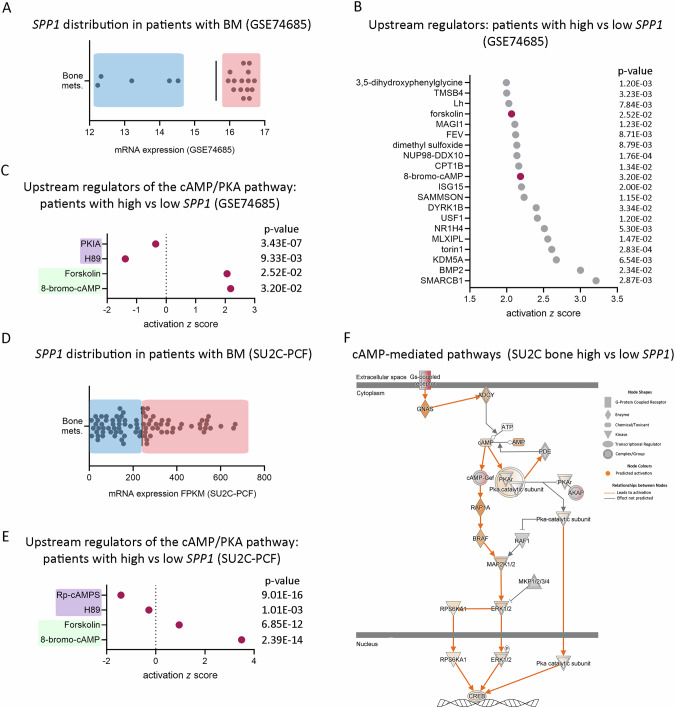
Clinical validation of the PKA/*SPP1* axis. **A** Dot plot depicting *SPP1* expression distribution in patients with bone metastasis from GSE74685 (*n* = 20). Mean expression was used to create two subpopulations with high (red) or low (blue) *SPP1* levels. **B** Dot plot showing the top 20 significantly activated upstream regulators and their *P* values obtained with Ingenuity Pathway Analysis (IPA) in patients with bone metastasis from GSE74685 with high *vs*. low *SPP1* levels. The x-axis represents the activation *z* score. Cherry dots represent PKA regulators. **C** Dot plot showing upstream/master regulators that activate (green) or inhibit (purple) PKA, and their *P* values obtained with IPA in patients with bone metastasis from GSE74685 with high *vs*. low *SPP1* levels. The x-axis represents the activation *z*-score. **D** Dot plot depicting *SPP1* expression distribution in patients with bone metastasis from the SU2C-PCF dataset (*n* = 83). Mean expression was used to create two subpopulations with high (red) or low (blue) *SPP1* levels. **E** Dot plot showing upstream/master regulators that activate (green) or inhibit (purple) PKA, and their *P* values obtained with IPA in patients with bone metastasis from the SU2C-PCF dataset with high *vs*. low *SPP1* levels. The x-axis represents the activation *z* score. **F** Representative cAMP/PKA pathway prediction in patients with bone metastasis from the SU2C-PCF dataset with high *vs*. low *SPP1* levels. Orange nodes and edges represent predicted activation.

Although overall *SPP1* levels are higher in human PCa BM samples from GSE74685, two distinct subpopulations of bone-metastatic PCa patients were identified with high and low *SPP1* expression (Fig. 4A), reflecting inter-patient PCa heterogeneity [12, 32]. An upstream regulation analysis (IPA) comparing these sub-populations revealed a positive activation z-score for PKA activators (8-bromo-cAMP and forskolin) (Fig. 4B, C) in BM samples with higher *SPP1* levels, while PKA inhibitors (PKIA and H89) exhibited a negative activation z-score (Fig. 4C). These results were validated in the SU2C-PCF dataset (Fig. 4D, E), directly associating high *SPP1* levels with active cAMP/PKA signaling in clinical samples (Fig. 4F**)**. We confirmed our results using a third dataset, including 22 primary PCa and 29 CRPC BM samples (GSE32269), where we observed that *SPP1* is significantly upregulated in the metastatic disease (Supplementary Fig. 6A). Consistently, patients with high *SPP1* levels (Supplementary Fig. 6B) displayed positive enrichment in PKA activators and negative enrichment in PKA inhibitors (Supplementary Fig. 6C) compared with patients with low *SPP1* levels.

### Implications of AR in PKA/*SPP1* axis modulation

**Fig. 5:**
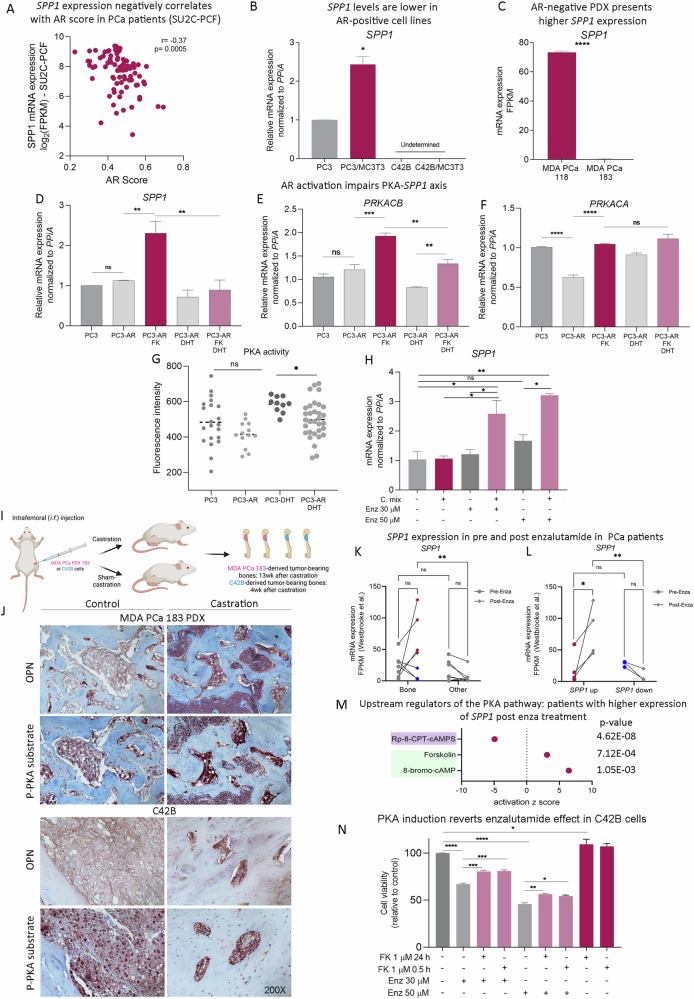
Crosstalk between the AR and PKA/*SPP1* axis. **A** Dispersion plot showcasing a Spearman correlation analysis between AR score and *SPP1* mRNA expression in patients with bone metastasis from the SU2C-PCF dataset (*n* = 83). **B** Gene expression levels of *SPP1* measured by RT-qPCR in PC3 grown alone (PC3) or co-cultured with MC3T3 (PC3/MC3T3) and in C42B grown alone (C42B) or co-cultured with MC3T3 (C42B/MC3T3). Values were normalized using *PPIA* as a reference gene and relativized to the expression in each control cell line. Statistical significance was assessed via Wilcoxon test. Three independent experiments were performed. Results are shown as the mean ± S.E.M. **C** *SPP1* expression (FPKM) assessed by RNA-seq in MDA PCa 118b and 183 growing intrafemorally (*n* = 5/group). Statistical significance was assessed using a Wilcoxon test. **D-F** *SPP1*, *PRKACB* and *PRKACA* gene expression levels assessed by RT-qPCR in PC3 cells transfected with an empty vector (PC3) or a plasmid to overexpress AR (PC3-AR) and treated or not with FK 1 μM and/or DHT 10 nM for 24 h. Values were normalized using *PPIA* as a reference gene and relativized to controls. Results are shown as mean ± S.E.M. Kruskal–Wallis was used to evaluate statistical significance. **G** PKA activity was measured by immunofluorescence in PC3 cells transfected with an empty vector (PC3) or a plasmid to overexpress AR (PC3-AR) and treated or not with DHT 10 nM for 24 h. Violin plots showcasing fluorescence intensity. Kruskal–Wallis was used to assess statistical significance. *P < 0.05. **H** *SPP1* mRNA expression levels in C42B cells growing in collagen and fibronectin coated surfaces (C. mix) and treated with enzalutamide (Enz) 30 or 50 µM for 96 h. Values were normalized using *PPIA* as a reference gene and relativized to controls. Results are shown as mean ± S.E.M. Kruskal–Wallis was used to evaluate statistical significance. **I** Schematic representation of intrafemoral (*i.f*.) implantation of MDA PCa 183 PDX or C42B-luc cells in male CB17 *SCID* mice, followed by surgical castration (or sham-castration as control). Tumor-bearing bones were harvested either 7 weeks or 4 weeks after castration, respectively, for further histological analysis. **J** Representative photomicrograph images of sections immunostained with OPN derived from MDA PCa 183 PDX or C42B-luc tumors growing *i.f*. in castrated or non-castrated (control) mice. Magnification 200X. **K** *SPP1* expression in patients with PCa bone or other metastasis from the Westbrook et al. dataset pre (circles) and post (rhombus) enzalutamide treatment. Paired *t* test was used to assess statistical significance. Paired *t* test was used to assess statistical significance. **L** *SPP1* expression in patients with PCa bone metastasis with high (cherry) or low (blue) *SPP1* levels from the Westbrook et al. dataset pre (circles) and post (rhombus) enzalutamide treatment. **M** Dot plot showing upstream/master regulators that activate (green) or inhibit (purple) PKA and their *P* values obtained with IPA in patients with bone metastasis from the Westbrook et al. dataset post *vs*. pre-enzalutamide, whose *SPP1* levels were increased after enzalutamide exposure. The x-axis represents the activation *z* score. **N** Cell viability assay in C42B cells pre-treated with forskolin (FK) 1 µM for 0.5 or 24 h and treated or not with enzalutamide 30 or 50 µM for 96 h. Kruskal–Wallis was used to evaluate statistical significance. Three independent experiments were performed. Statistical significance was set at *P* < 0.05. *P < 0.05, **P < 0.001, ***P < 0.0001, ***P < 0.00001. ns not significant.

Previous reports indicate that AR pathway can negatively impact PKA signaling [40]. In line with that, *SPP1* mRNA levels inversely correlated with AR score in BM samples from the SU2C-PCF dataset (Fig. 5A). Thus, differential response on *SPP1* induction could be a consequence of different levels of AR activity among BM samples. To further explore the relation between PKA/*SPP1* and AR status we characterized bone-induced *SPP1* expression in models with different AR statuses/responsiveness, both in vitro and in vivo. By comparing *SPP1* mRNA levels in PC3 (AR-) and C42B (AR+) cell lines, we observed that C42B cells did not present detectable *SPP1* expression even when co-cultured with bone progenitors (Fig. 5B). Moreover, we performed pre-clinical studies using relevant PDX models such as MDA PCa 183-A and 118b, both derived from BM. 183-A is an AR+ tumor derived from a treatment-naive patient, and 118b is an AR- CRPC. Consistently, we observed high *SPP1* levels in 118b whereas its expression was barely detectable in 183-A when grown *i.f*. in CB17 *SCID* mice (Fig. 5C, Supplementary Fig. 7A). Of note, in both cases tumor-bearing bones displayed upregulated *Col1a1* (Supplementary Fig. 7B) and *Fn1* (Supplementary Fig. 7C) levels compared with non-tumor-bearing bones, suggesting that differential response is due to intrinsic characteristics of each model, possibly influenced by AR signaling status.

To directly examine the interaction between AR and the PKA/*SPP1* pathway, we transfected AR-negative PC3 cells with a human AR expression vector. AR expression was confirmed by RT-qPCR and Western blot, and AR activity was validated using the PSA-Luc reporter plasmid following DHT treatment (10 nM) and PSA expression levels by RT-qPCR (*KLK3* gene; Supplementary Fig. 7D–F). FK-mediated *SPP1* and *PRKACB* (PKA catalytic subunit beta) overexpression was prevented by DHT treatment in PC3 cells expressing AR (Fig. 5D, E). Interestingly, AR-expressing PC3 cells showcased significantly lower basal levels of *PRKACA* (PKA catalytic subunit alfa) compared to control (Fig. 5F), and no differences in FK and DHT treated cells were observed. Moreover, PC3-AR cells treated with DHT displayed lower levels of PKA activity (Fig. 5G). Accordingly, *SPP1* expression was induced in C42B cells growing in collagen/fibronectin coated surfaces only upon AR-signaling inhibition with enzalutamide (30 or 50 µM, 96 h; Fig. 5H).

In vivo, we injected MDA PCa PDX 183-A or C42B-luc cells into the distal end of femurs of 6- to 8-wk-old male CB17 *SCID* mice. After a month, mice were randomized into sham-castrated (control) or surgically castrated groups (Fig. 5I). Consistently, we observed an increase in both OPN staining and PKA activity by IHC either in MDA PCa 183-A or in C42B xenotransplants growing *i.f*. after castration (Fig. 5J). These results underscore the crucial role of AR activity in regulating the PKA/*SPP1* axis. To clinically assess whether AR signaling impacts the PKA/*SPP1* axis in PCa bone metastasis, we explored the Westbrooke et al. dataset [34] containing longitudinal samples of metastatic PCa before and after enzalutamide treatment. In this dataset BM samples also showed increased *SPP1* expression compared with other metastatic sites only after enzalutamide treatment (Fig. 5K). In line with previous observations we also identified two sub-populations among BM samples, exhibiting or not *SPP1* induction after enzalutamide treatment (Fig. 5L). Accordingly, the activation z-score of cAMP/PKA signaling regulators suggests pathway activation in patients with *SPP1* induction after enzalutamide treatment (Fig. 5M), while the remaining patients displayed increased activation z-score for the PKA inhibitor, PKIA (Supplementary Fig. 7G). Thus, indicating that in a subpopulation of patients the activation of this mechanism is the result of tumor extrinsic (microenvironment) and intrinsic (AR signaling) factors. These results are in line with previous reports indicating that androgen deprivation therapy (ADT) increases cAMP/PKA/CREB signaling, and implicated this axis with PCa transition to neuroendocrine disease [40], associated with treatment resistance and disease progression [40]. In this sense, we observed a protective effect of PKA pre-activation with FK in C42B cells against the cytotoxic effect of enzalutamide (Fig. 5N), confirming the role of PKA in modulating AR-targeted therapies response.

## Discussion

PCa is recognized by the inter-patient heterogeneity in treatment response and clinical outcome, which is not well understood. Contributing factors to this heterogeneity include tumors’ genomic background, host determinants and the tumor microenvironment [3]. CRPC progression is commonly associated with the development of BM, a milestone that heralds the transition to lethal PCa [3, 41]. In this study, we identify a novel bone–tumor interaction axis in which the BME modulates PKA signaling, promoting *SPP1*/OPN expression in PCa cells. This mechanism may contribute to adaptive responses in bone and affect sensitivity to AR-targeted therapies. Accordingly, we propose that *SPP1*/OPN serves as a biomarker for PCa tumors with active PKA signaling, with potential implications for patient stratification and therapy selection.

When PCa colonizes the bone niche, a strategy adopted by tumor cells to fully exploit the bone metastatic niche relies on their ability to acquire an osteoblast-like phenotype, also known as osteomimicry [42]. PCa cells in the bone start to express bone matrix proteins, including OPN as shown in this work, and thus modulating bone-tumor cell crosstalk favoring tumor progression [42]. High OPN levels in tumor tissue and plasma have been associated with poor prognosis in both BCa and PCa [7, 43], but the environmental cues that regulate its expression in bone remain insufficiently understood. Among all the osteoblast-derived molecules released by tumor cells, Runx2 has been suggested as a regulator of OPN expression [44], however the microenvironmental factors driving this mechanism are poorly described. This study sheds light on a novel mechanism underlying OPN overexpression in PCa tumors. We demonstrate that type I collagen and fibronectin — two abundant ECM proteins in bone—are capable of inducing PKA activation and *SPP1* expression in PCa cells.

Type I collagen can engage α2β1 integrins and discoidin domain receptors (DDR1/DDR2), while fibronectin binds α5β1 and αvβ3 integrins—receptors known to activate intracellular signaling cascades including FAK, Src [45, 46], and potentially cAMP/PKA pathways. Although we did not directly identify the specific receptors mediating PKA activation in our model, these findings establish a functional link between bone ECM components and tumor signaling.

In parallel, our analysis of the *SPP1* promoter revealed CREB/CREBP1 binding motifs with high similarity scores, suggesting that PKA may regulate *SPP1* transcription via CREB phosphorylation and nuclear translocation. This model is consistent with the nuclear localization of phosphorylated PKA substrates observed by immunofluorescence and provides a testable hypothesis for future mechanistic studies.

The regulatory influence of PKA modulation on cellular proliferation is well documented across various cancer types [47]. Notably, a recent investigation in BCa demonstrated that the PKA inhibitor H89 exerted a robust antitumor response in vivo, mediated by alterations in the immune microenvironment [48].

While OPN upregulation is prevalent in BCa BM (83% of cases), it manifests in only a limited subset (11%) of PCa patients [12], which is consistent with our observations regarding disease heterogeneity. In this context, our findings reveal that AR signaling influences the PKA/*SPP1* axis. These results are in line with prior studies reporting increased cAMP levels and PKA activity in PCa following treatment with AR signaling inhibitors (ARSI), correlating with aggressive disease [49, 50]. Our results support the role of PKA activation as a potential mechanism of progression to ARSI and the inclusion of *SPP1*/OPN in a biomarker-driven strategy for patient care.

In conclusion, we propose a model wherein the BME promotes PCa progression via PKA activation, positioning *SPP1*/OPN as a promising biomarker for BM with PKA activation.

## Supplementary information

Supplementary information

## Data Availability

The datasets generated during and/or analyzed during the current study are available from the corresponding author on reasonable request.
