# Supplementary material for: PKA-driven SPP1 activation as a novel mechanism connecting the bone microenvironment to prostate cancer progression
Source: Oncogene. 2025 Aug 2;44(38):3568–79. doi: 10.1038/s41388-025-03511-z (PMC12436191; doi:10.1038/s41388-025-03511-z)
Supplement: Supplementary file 1 — Supplementary information [file 41388_2025_3511_MOESM1_ESM.pdf]

Supplementary Methods

Secretome analysis by mass spectrometry. To analyze/detect proteins secreted by both tumor and bone cells into the culture medium, we conducted a proteomic study of the conditioned media (CM) derived from the co-culture of PC3 cells and MC3T3 cells. After completing the co-culture experiment, the CM from each experimental condition was collected, centrifuged (10 min, 2000 rpm), and transferred to a clean tube to remove cells and residual debris. The media were then concentrated using centrifugal columns (Vivaspin® 20 Polyethersulfone; cut-off >10 kDa; #VS2001; Sartorius, Argentina). Once concentrated, the media were washed with PBS (3 times) in the same columns, and the protein content was quantified using the BCA assay. For protein identification, the CM was subjected to protein digestion and mass spectrometry analysis, performed at the CEQUIBIEM Proteomics Center, University of Buenos Aires/CONICET, following this protocol: Protein samples were reduced with dithiothreitol (DTT) in 50 mM ammonium bicarbonate at a final concentration of 10 mM (45 min, 56°C) and alkylated with iodoacetamide in the same solvent at a final concentration of 20 mM (40 min, room temperature, in darkness). Protein samples were digested with Trypsin (Promega V5111) overnight. Peptides were extracted and desalted using C18 zip tips (Merck Millipore). The digested samples were analyzed by nanoLC-MS/MS using a QExactive Mass Spectrometer (Thermo Scientific) coupled to an EASY-nLC 1000 nanoHPLC (Thermo Scientific). For LC-MS/MS analysis, 2 µg of peptides were injected into a reverse-phase column (C18, 2 µm, 100 Å, 50 µm x 150 mm) Easy-Spray Column PepMap RSLC (P/N ES801), suitable for high-resolution protein complex separation. A 120-minute run was performed at a flow rate of 300 nL/min with a gradient from 7% solvent B (5 min) to 35% (120 min). Solvent A consisted of 0.1% formic acid in water, and solvent B consisted of 0.1% formic acid in acetonitrile. The injection volume was 2 µL. A voltage of 3.5 kV was used for Electrospray Ionization (Thermo Scientific, EASY-SPRAY). The MS equipment included a high-energy collision dissociation (HCD) cell for fragmentation and an Orbitrap analyzer (Thermo Scientific, Q-Exactive). Data acquisition was performed using the XCalibur 3.0.63 software (Thermo Scientific), with the system configured to identify peptides simultaneously with chromatographic separation. Full-scan mass spectra were acquired in the Orbitrap analyzer. The mass range scanned was 400–1800 m/z, with a resolution of 70,000 at 400 m/z, and the twelve most intense ions in each cycle were subsequently isolated, fragmented by HCD, and measured in the Orbitrap analyzer. Peptides with a +1 charge or unassigned charge states were excluded from fragmentation for MS2. We have included this exact explanation as a methodology in the Supplemental Methods section.

SPP1 **promoter sequence analysis**.

TFBind webtool[1] was used for searching CREB/CREBP binding sites within the SPP1 promoter region. First, we conducted a search in NCBI Gene database for human SPP1 (Gene ID: 6696) and the genomic regions within its sequence. We selected and downloaded the FASTA for the sequenced annotated as promoter (NC_000004.12:87973468-87975710) and proceeded to analyze it using TFbind. Briefly, this webtool is designed to predict transcription factor binding sites (from TRANSFAC R3.4 matrix[2]) within a DNA sequence, returning a similarity score to the consensus motif (0-1 score). This tool returns only those transcription factor binding sites with a similarity score above the cut-off values calculated by Tsunoda and Takagi [1]. Only motifs for CREB/CREBP1 with a similarity score above 0.8 are shown. The sequence logos were plotted using ggseqlogo package[3].

Supplementary Tables

Supplementary Table 1. Top 10 upregulated (red) and downregulated (blue) genes in bone metastasis *vs*. primary tumors from the GSE74685 dataset. Differential expression analysis was performed using the limma package in R, with Benjamini-Hochberg correction (adj.P.Val).

Gene	logFC	adj.P.Val
FABP4	0.53251459	2.3624E-08
COL11A1	0.50709994	4.5827E-06
SPP1	0.50018156	4.6878E-06
DEFA3	0.4997894	0.00010425
IBSP	0.48117746	1.9637E-08
UBE2E1	0.46916114	1.3977E-11
HBA2	0.4549175	2.0769E-05
HBD	0.44366908	0.00014044
HBA1	0.43214515	2.1127E-05
APP	0.42766319	2.0818E-09
MAOB	-0.58344031	1.9897E-07
MLLT1	-0.57069282	2.8869E-14
DACT2	-0.5552873	0.00073756
CCDC144A	-0.54085587	3.3145E-11
SYT1	-0.53167898	1.7374E-05
SERPINA6	-0.52717586	2.5246E-11
SLC6A17	-0.52626893	1.3247E-11
MYH11	-0.52223974	2.9805E-05
LGSN	-0.5098587	5.1238E-06
APCS	-0.50641258	2.1265E-14

Supplementary Figures

Supplementary Figure 1

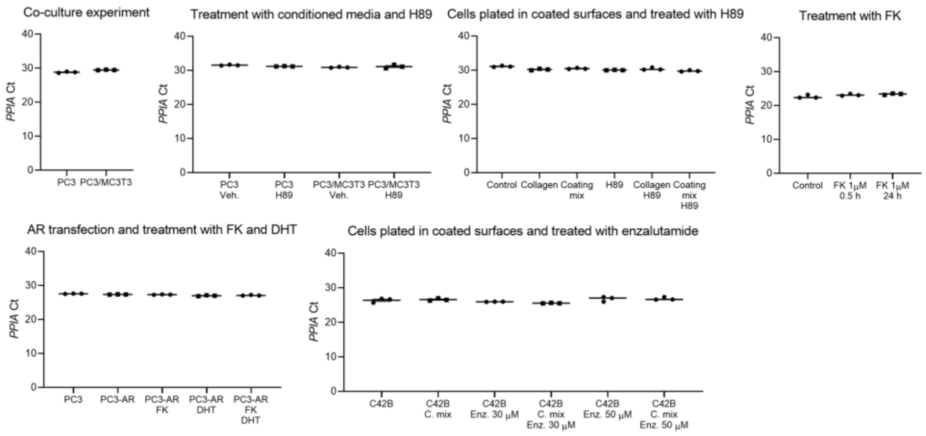

SUPPLEMENTARY FIGURE 1. *PPIA* expression levels assesed by RT-qPCR in different experimental conditions evaluted in this work: PC3 cells co-cultured with bone progenitors, treated with conditioned media and/or H89, plate in collagen and/or fibronectin enriched surfaces, treated with forskolin (FK), and transfected with AR and treated with FK and/or dihydrotestosterone (DHT), and in C42B cells plated in collagen and fibronectin enriched surfaces and treated with enzalutamide.

Supplementary Figure 2

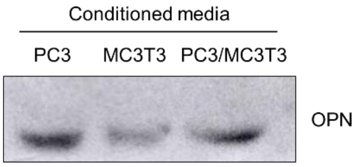

SUPPLEMENTARY FIGURE 2. OPN expression levels in conditioned media (CM) from PC3, MC3T3 and PC3/MC3T3. CMs were collected, centrifuged (2000 rpm, 10 min), and concentrated by centrifugation (3 × 15 min, 6500 g) in VivaSpin® tubes (vivaspin® 20 Polyethersulfone; cut off > 10 kDa; #VS2001; Sartorius, Germany), and OPN was detected by Western blotting.

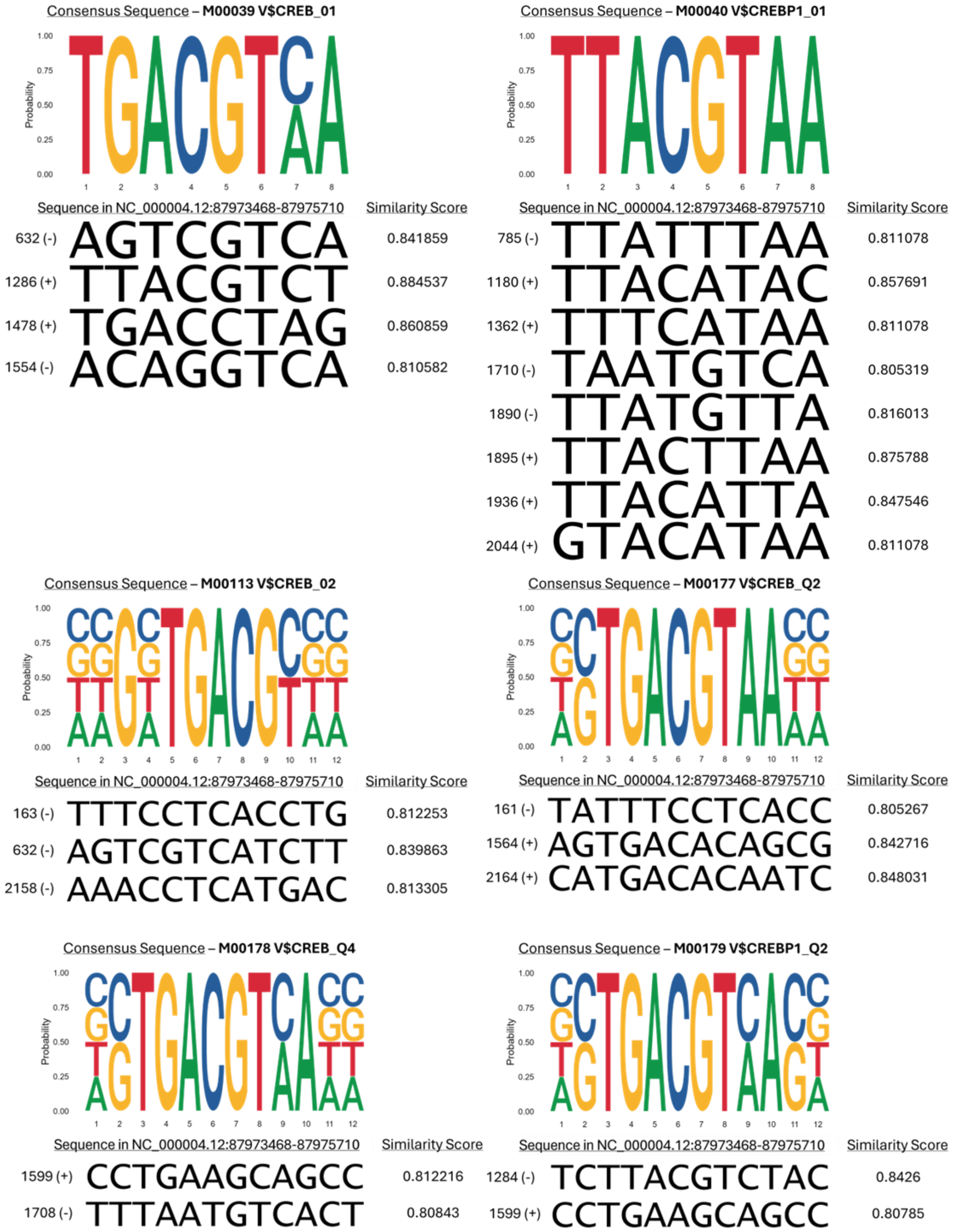

SUPPLEMENTARY FIGURE 3. Sequence logo representation of the consensus and identified motifs in the *SPP1* promoter. The sequence logo displays the nucleotide composition of the consensus motif for CREB and CREBP (top) and the in silico identified motifs within the *SPP1* promoter (bottom). More than one nucleotide is represented in the consensus sequence for degenerate positions. The x-axis represents the position within the motif. The *SPP1* promoter sequence NC_000004.12:87973468-87975710 was obtained from NCBI. The analysis was performed using Tfbind.hgc.jp, which uses TRANSFAC R3.4. consensus motifs and calculates similarity scores. The similarity score threshold was set above 0.8.

Supplementary Figure 4

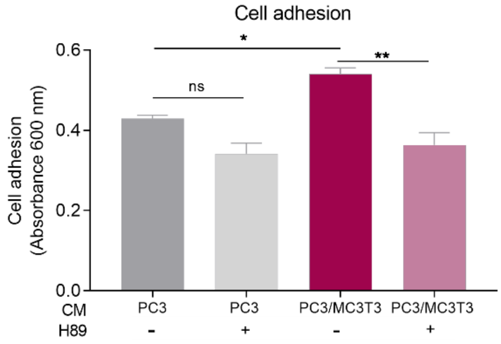

SUPPLEMENTARY FIGURE 4. PC3 cells were cultured in the conditioned media (CM) of PC3 or PC3/MC3T3 co-culture for 24 h, and with or without the addition of the PKA inhibitor, H89 (10 μM), during the last 3 h of culture. After 24 h, cells were harvested and plated at a density of 50000 cells/well in a 96 multiwell plate for 1 h, washed with PBS and stained with crystal violet. Crystal violet was extracted with 10% acetic acid (V/V), and quantification of absorbance at 600 nm was performed. Kruskal-Wallis test was used to assess statistical differences. Statistical differences: *P<0.05, **P<0.01, ns: not significant.

Supplementary Figure 5

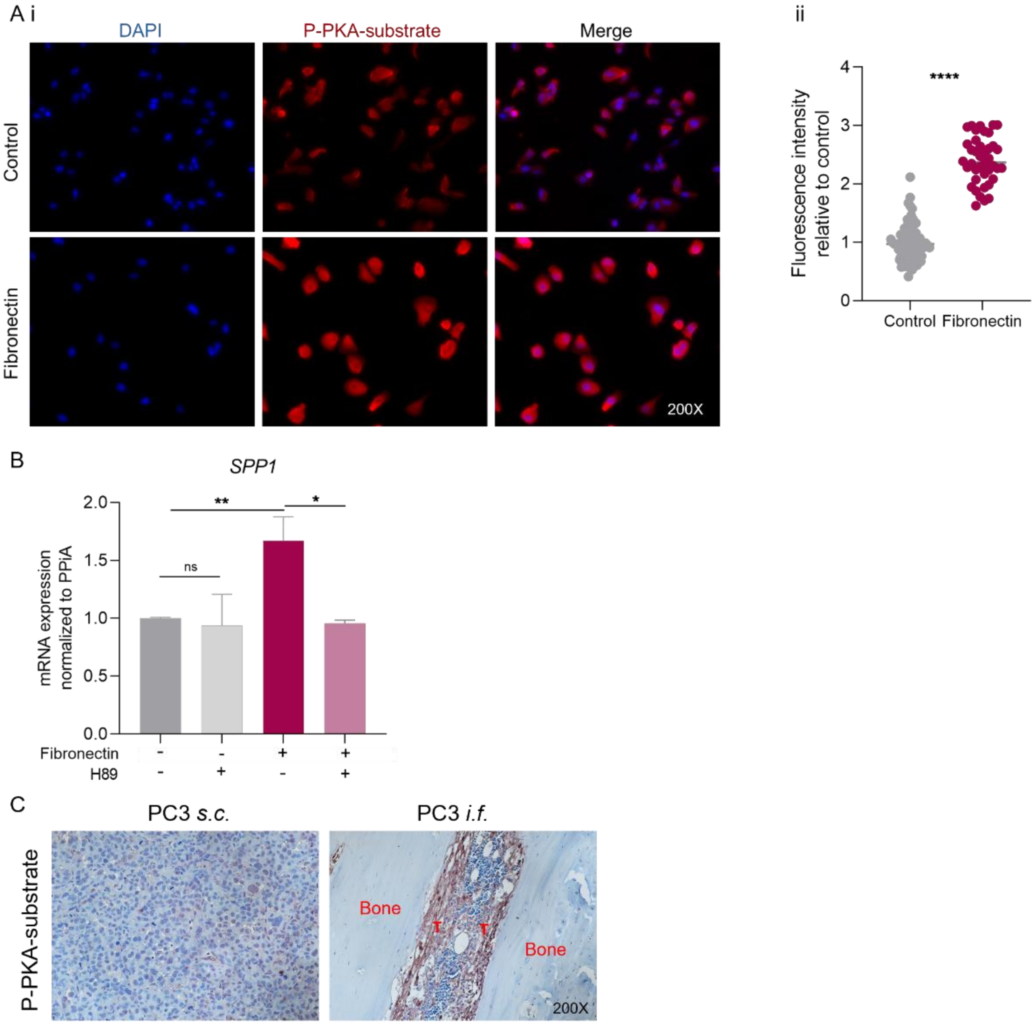

SUPPLEMENTARY FIGURE 5. A) Immunofluorescence staining and epifluorescence microscopy analysis for p-PKA substrate (i) and fluorescence intensity quantification (ii) in PC3 cells seeded on fibronectin or control. **B)** *SPP1* gene expression levels evaluated by RT-qPCR in PC3 cells cultured for 24 h in wells coated with fibronectin, with or without H89 (10 μM) added during the last 3 h of culture. PC3 cells seeded in untreated wells were considered controls. Values were normalized using *PPIA* as a reference gene and relativized to controls. Kruskal-Wallis was used to evaluate statistical significance. Results are shown as mean ± S.E.M. Statistical significance was set at $P < 0.05$. * $P < 0.05$, ** $P < 0.001$, ***$P < 0.0001$. **C)** Representative photomicrograph image of PC3 tumors *s.c.* and *i.f.* sections immunostained with anti-pPKA substrate (n=5). Magnification 200X. T = Tumor.

Supplementary Figure 6

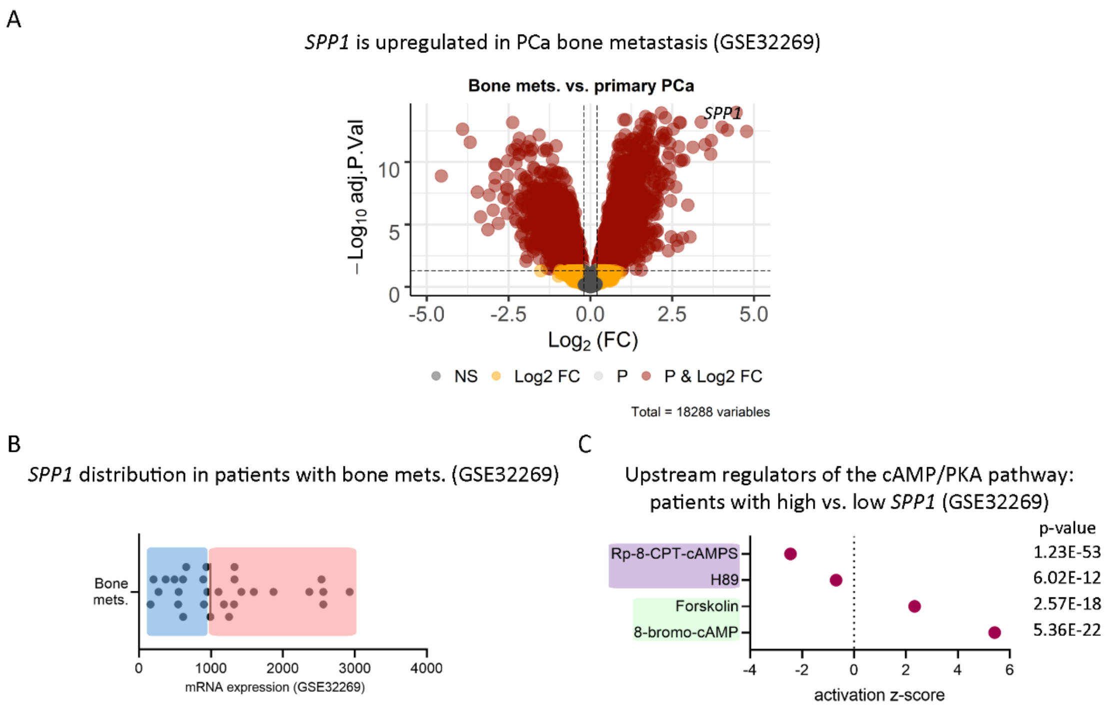

SUPPLEMENTARY FIGURE 6. A) Volcano plot depicting differential gene expression analysis of the GSE32269 dataset comparing bone metastases (n=29) *vs.* primary PCa (n=22) samples. Significant differential expression ($P < 0.05$, $|\text{Log}_2 \text{ fold change}| > 0.2$) is represented for each comparison as cherry dots. Wilcoxon *t* test was used to calculate statistical significance. NS: not significant; Log_2FC: Log_2 fold change. Dark grey dots: $|\text{Log}_2 \text{ fold change}| < 1$ and $P > 0.05$; yellow dots: $|\text{Log}_2 \text{ fold change}| > 1$ and $P > 0.05$; light grey dots: $|\text{Log}_2 \text{ fold change}| < 1$ and $P < 0.05$; cherry dots: $|\text{Log}_2 \text{ fold change}| > 1$ and $P < 0.05$. **B)** Dot plot depicting *SPP1* expression distribution in patients with bone metastasis from GSE32269 (n=29). **C)** Dot plot showing upstream/master regulators that activate (green) or inhibit (purple) PKA, and their *P* values obtained with IPA in patients with bone metastasis from GSE32269 with high *vs.* low *SPP1* levels. The x-axis represents the activation *z*-score.

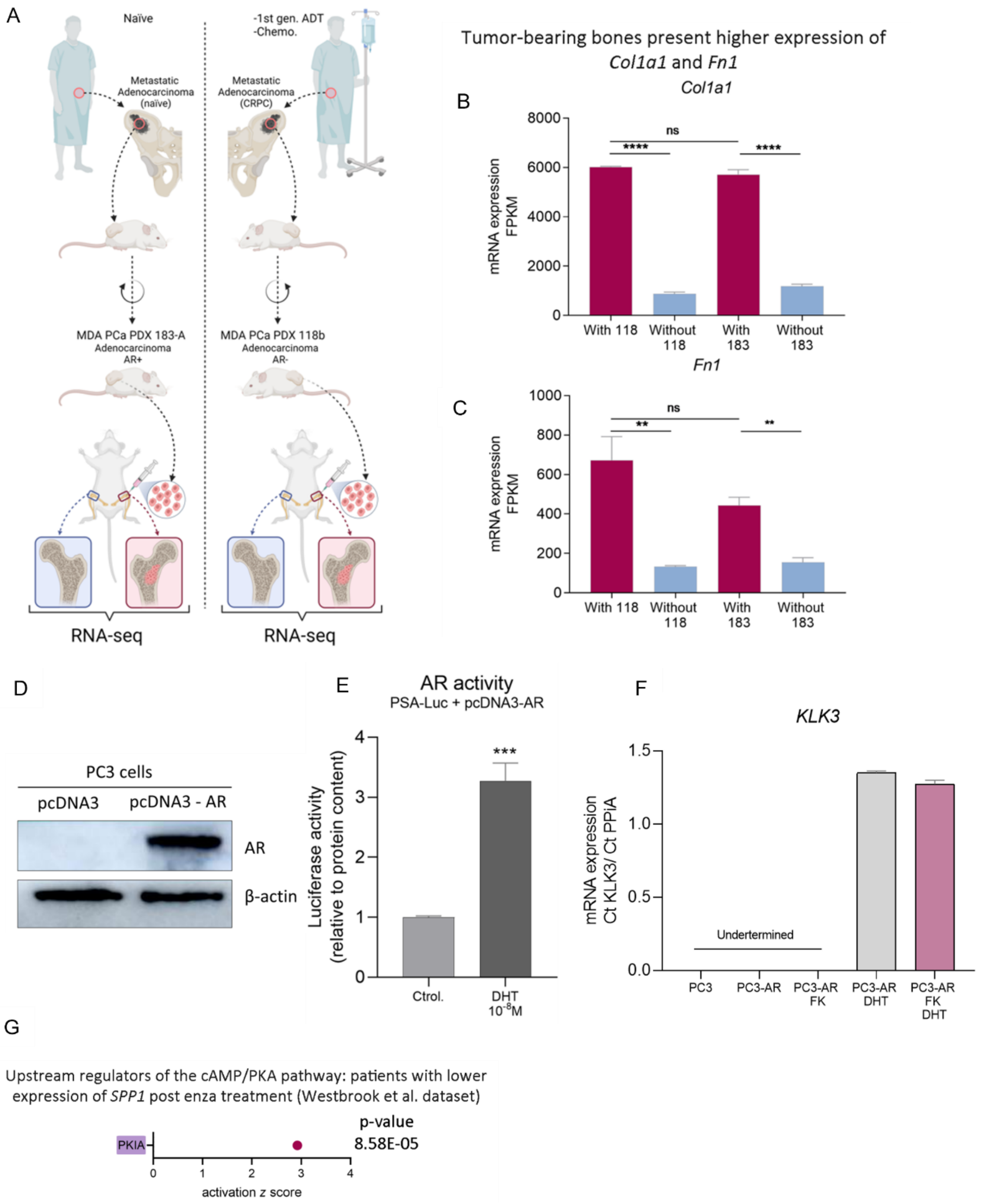

SUPPLEMENTARY FIGURE 7. A) Schematic representation of MDA PCa 183-A and 118b PDX characteristics, and intrafemoral injection experimental designs. **B-C)** Bar plots depicting gene expression (FPKM) values for *Col1a1* **(E)** and *Fn1* **(F)** in MDA PCa 118b and 183-A tumor-bearing and non-tumor-bearing bones. Kruskal-Wallis was used to assess statistical significance. Expression values for each gene were normalized using *PPIA* as a reference gene, and expression in tumor-bearing bones was relativized to expression in non-tumor-bearing bones. **D)** AR protein levels in PC3 cells transfected with pcDNA3 or pcDNA3-AR. Total proteins were

extracted, and AR expression was determined by Western Blot. **E)** Luciferase activity relative to total protein in PC3 cells co-transfected with a PSA-Luc reporter vector and pcDNA3-AR, treated or not with 10 nM DHT. **F)** *KLK3* gene expression levels assessed by RT-qPCR in PC3 cells transfected with an empty vector (PC3) or a plasmid to overexpress AR (PC3-AR) and treated or not with FK 1 μM and/or DHT 10 nM for 24 h. Values were normalized using *PPIA* as a reference gene. The mean ± SD of three independent experiments is plotted. Significant differences: ***P<0.0001.**G)** Dot plot showing upstream/master regulator that inhibit PKA and its *P* values obtained with IPA in patients with bone metastasis from the Westbrook et al. dataset post vs pre enzalutamide, whose *SPP1* levels were lower after enzalutamide exposure. The x-axis represents the activation *z* score.

References

1 Tsunoda T, Takagi T. Estimating transcription factor bindability on DNA. *Bioinformatics* 1999; **15**: 622–630.

2 Wingender E, Dietze P, Karas H, Knüppel R. TRANSFAC: a database on transcription factors and their DNA binding sites. *Nucleic Acids Res* 1996; **24**: 238–241.

3 Wagih O. ggseqlogo: a versatile R package for drawing sequence logos. *Bioinformatics* 2017; **33**: 3645–3647.
